# Prediction of Targets and Mechanisms of Top Ten Core “Food–Medicine Homologous Traditional Chinese Medicines” in Delaying Vascular Aging: An Integrative Computational Study

**DOI:** 10.3390/ph19010131

**Published:** 2026-01-12

**Authors:** Yiling Bai, Qian Liu, Qing Zhou, Pengyang Xiao, Lina Xia

**Affiliations:** School of Health Preservation and Rehabilitation, Chengdu University of Traditional Chinese Medicine, Chengdu 610075, China; baiyiling95@163.com (Y.B.);

**Keywords:** “food–medicine homologous traditional Chinese medicines”, human-origin vascular aging, inverse network pharmacology, bioinformatics, molecular docking, molecular dynamics simulation

## Abstract

**Background and Objectives**: Many “food–medicine homologous traditional Chinese medicines (TCMs)” have been shown to delay vascular aging. In this study, we will select “food–medicine homologous TCMs” with the most potential to delay human-origin vascular aging and predict their core targets and mechanisms. **Methods**: Human-origin vascular-aging-related genes were screened from the NCBI and Aging Atlas databases. Candidate “food–medicine homologous TCMs” were initially filtered by constructing a protein–protein interaction network, followed by Gene Ontology and Kyoto Encyclopedia of Genes and Genomes enrichment analyses. Key targets were validated in the Gene Expression Omnibus database and further screened by least absolute shrinkage and a selection operator. Finally, molecular docking and molecular dynamics simulations identified core targets. **Results**: Ten core “food–medicine homologous TCMs” with potential to delay human-derived vascular aging were identified: *Crocus Sativus* L., *Glycyrrhiza uralensis Fisch.*, *Chrysanthemum morifolium Ramat.*, *Astragalus membranaceus* (*Fisch.*) *Bunge*, *Sophora japonica* L., *Hippophae rhamnoides* L., *Portulaca oleracea* L., *Lonicera japonica Thunb.*, *Citrus aurantium* L. *var. amara Engl.*, and *Morus alba* L. Further analysis indicated that β-Carotene within these core “food–medicine homologous TCMs” may represent a potential active component targeting matrix metalloproteinase-1, with its action potentially linked to the interleukin-17 signaling pathway. The present study highlights the new hypothesis that immunosenescence (Th17/IL-17) is involved in vascular aging, suggesting that the top ten core “food–medicine homologous TCMs” may delay vascular aging by regulating immune cell function. **Conclusions**: The top ten “food–medicine homologous TCMs” provide potential candidates for functional products that delay vascular aging and provide computationally predicted mechanistic insights and a scientific basis for novel therapies.

## 1. Introduction

With the accelerated aging of the world’s population, age-related diseases have become the focus of current research and attention. Among these, vascular-related diseases have a very high prevalence in the elderly population [1,2]. In particular, vascular aging is a high-risk factor for the development of vascular-related diseases, including atherosclerosis, hypertension, and peripheral artery disease [2]. Therefore, applying the correct methods to delay vascular aging is of great significance in preventing and controlling chronic diseases in the elderly and coping with the increasingly serious problem of population aging. The existing research has established that commonly prescribed clinical medications for managing cardiovascular risk factors—such as angiotensin-converting enzyme inhibitors, statins, and hypoglycemic agents—can to some extent improve vascular function and delay vascular aging. However, no targeted drugs have yet been identified that act specifically on the core regulatory targets of vascular aging [3].

In recent years, the advantages of TCMs in preventing vascular senescence have gradually emerged, playing an important role in protecting vascular endothelial function and maintaining vascular structure [4]. Among various TCMs, those classified as “food–medicine homologous TCMs” have attracted attention for their dual nutritional and medicinal value. The term “food–medicine homologous TCMs” refers to Chinese medicines that can be used as both food and medicine, with clear efficacy, low side effects, and flexible dosage forms [5]. Through scientific formulation design and process optimization, extracting active constituents from “food–medicine homologous TCMs” with foodstuffs and applying them to food products enables the development of functional foods with specific health benefits, thereby providing natural sources for the functional components of such foods. Modern research has confirmed that a variety of “food–medicine homologous TCMs” have the effect of mitigating vascular aging. There is evidence [6] that *Polygonati rhizoma* in aged rat endothelial progenitor cells (EPCs) can slow down the senescence process of EPCs in passaged cultures by reducing the level of reactive oxygen species (ROS) and protecting the function of EPCs. In addition, Astragalus polysaccharides [7] can promote autophagosome formation through microtubule-associated protein 1 light chain 3 (LC3)-sequestosome-1 (p62)-autophagy-related protein 7 (Atg7) and ensure autophagic flux, which may be one of the mechanisms by which it delays the aging of vascular endothelial cells (ECs). However, the identification of core “food–medicine homologous TCMs” with significant potential to delay vascular aging remains unexplored, motivating the present study.

Reverse network pharmacology is used to establish a “disease–target–drug” reverse screening methodology. This approach overcomes the limitations of traditional network pharmacology, which relies heavily on existing knowledge and faces constraints in screening scope. It enables direct identification of potential active drugs that are precisely linked to core disease targets, thereby enhancing screening specificity. Concurrently, the integration of multiple computational methods—including the least absolute shrinkage and selection operator (LASSO) machine learning algorithm, molecular docking, and molecular dynamics simulations—reinforces the reliability of outcomes. This screening method combines comprehensiveness with precision.

This study employs reverse network pharmacology [8]; it is proposed to screen the core TCMs for delaying human-origin vascular aging, based on the concept of “disease target–active components–homology of medicine and food.” Combined Gene Ontology (GO) and Kyoto Encyclopedia of Genes and Genomes (KEGG) enrichment analyses predicted the mechanism of action of these core TCMs in delaying human-origin vascular aging. The Gene Expression Omnibus (GEO) database also verified the correctness of the human-origin vascular aging targets, and the core targets were further screened using the LASSO machine learning. Finally, molecular docking techniques and molecular dynamics simulations were used to predict the binding activities of the major active components of the core TCMs to human-origin vascular aging core targets [9].

In this study, we used the integration of reverse network pharmacology, bioinformatics, machine learning, molecular docking, and molecular dynamics simulation to systematically screen the core “food–medicine homologous TCMs” that delay vascular aging for the first time and predict the core targets and mechanisms of action.

## 2. Results

### 2.1. Screening of 54 Genes Related to Human-Origin Vascular Aging-Associated Genes

**Figure 1:**
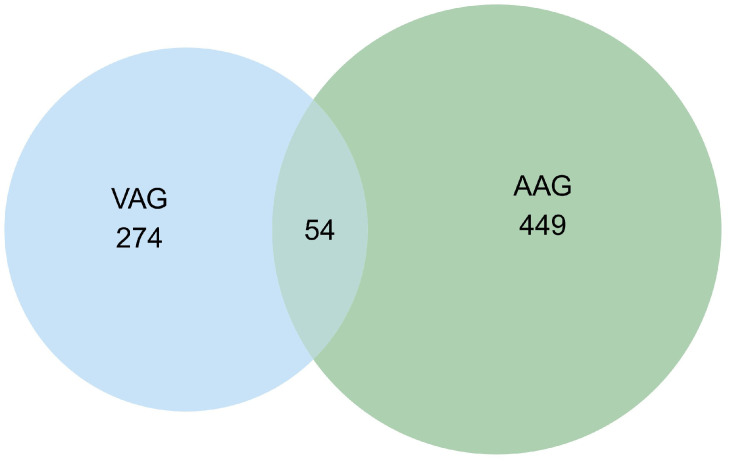
Identification of human-origin vascular aging-associated genes, based on VAG and AAG Venn diagrams.

In this study, we obtained 328 human-origin vascular-associated genes (VAGs) from the National Center for Biotechnology Information (NCBI) gene database, using “vasculature” and “Homo sapiens” as search terms. Subsequently, 503 human-origin aging-associated genes (AAGs) were obtained from the Aging Atlas. The 328 human-origin VAGs and 503 human-origin AAGs were pooled, and 54 human-origin vascular aging-associated genes were finally screened to plot a Venn diagram (Figure 1). These 54 human-origin vascular aging-associated genes serve as targets for subsequent studies. The human-origin vascular aging-associated genes are listed in Supplementary Table S1.

### 2.2. A Total of 39 Targets May Be Key Targets of Human-Origin Vascular Aging

**Figure 2:**
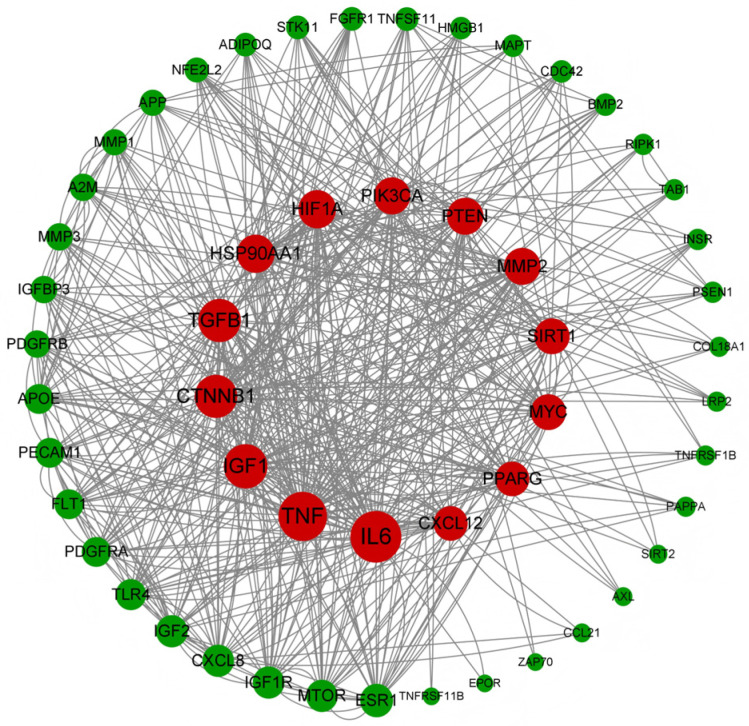
PPI map of human-origin vascular aging key targets.

The 54 human-origin vascular aging-associated genes were imported into the Search Tool for the Retrieval of Interacting Genes/Proteins (STRING) database for protein–protein interaction (PPI) network construction, which was found to contain 54 nodes and 267 edges, with an average node degree value of approximately 9.89, with the help of the software Cytoscape 3.8.0 for visualization and the CytoNCA plug-in for network topology analysis. The size of the circular nodes is sorted according to the degree value, and the larger degree value indicates a more critical role for the target point in this PPI network (Figure 2). A total of 39 nodes with node degree values above the mean value of 9.89 were utilized as the key targets in this study, among which targets such as interleukin-6 (IL-6), tumor necrosis factor (TNF), insulin-like growth factor 1 (IGF1), β-catenin (CTNNβ1), and transforming growth factor β1 (TGFβ1) may be the key targets of human-origin vascular aging. Specific information on the core targets is provided in the table (Table 1).

### 2.3. Identifying the Top Ten Core “Food–Medicine Homologous TCMs” for Delaying Human-Origin Vascular Aging

**Figure 3:**
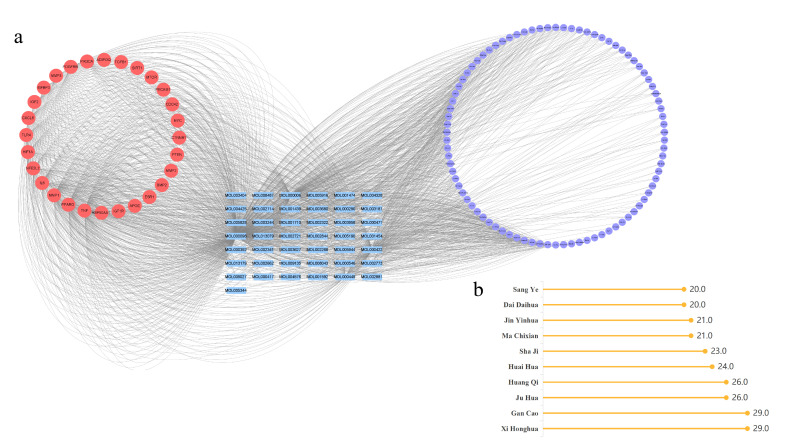
Screening results of “food–medicine homologous TCMs” for delaying human-origin vascular aging. (**a**) Network diagram of “human-origin vascular aging core targets–active compounds–food–medicine homologous TCMs”. (**b**) Top ten core “food–medicine homologous TCMs” with the highest degree values.

Cytoscape 3.8.0 software was used to construct a network diagram of “human-origin vascular aging core targets–active compounds–food–medicine homologous TCMs,” in which the red nodes represented the human-origin vascular aging core targets, the blue nodes represented the active compounds, and the purple nodes represented “food–medicine homologous TCMs” (Figure 3a). Twenty-eight of the 39 human-origin vascular aging core targets can be reverse-matched to 43 active compounds, corresponding to 86 “food–medicine homologous TCMs”. The CytoNCA plugin was used to calculate the degree value of the “food–medicine homologous TCMs” node, with a higher degree value representing a more central node. The top ten “food–medicine homologous TCMs” with the highest degree values were selected for further study, including Xi Honghua (*Crocus sativus* L.), Gan Cao (*Glycyrrhiza uralensis Fisch.*), Ju Hua (*Chrysanthemum morifolium Ramat.*), Huang Qi (*Astragalus membranaceus* (*Fisch.*) *Bunge*), Huai Hua (*Sophora Japonica* L.), Sha Ji (*Hippophae rhamnoides* L.), Ma Chixian (*Portulaca oleracea* L.), Jin Yinhua (*Lonicera japonica Thunb*.), Dai Daihua (*Citrus aurantium* L. *var. amara Engl.*), and Sang Ye (*Morus alba* L.) (Figure 3b). The specific degree values for the “food–medicine homologous TCMs” are detailed in Supplementary Table S2. In this study, the top ten core “food–medicine homologous TCMs” were used as candidates for subsequent studies.

### 2.4. Top Ten Core “Food–Medicine Homologous TCMs” May Delay Human-Origin Vascular Aging by Acting on Targets Such as TNF

**Figure 4:**
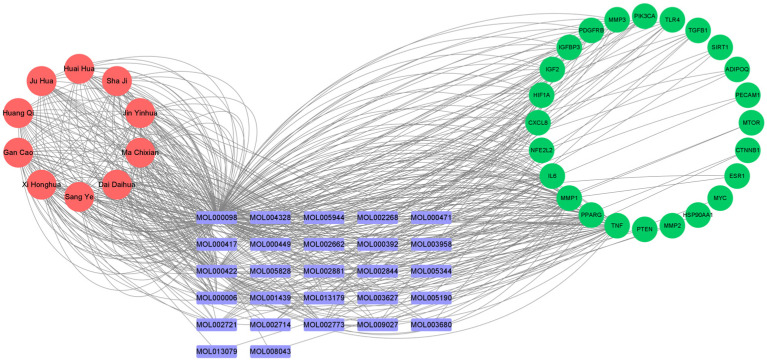
Network diagram of “core food–medicine homologous TCMs–active compounds–human-origin vascular aging targets”.

Cytoscape software was used to extract the sub-networks of the above top ten core TCMs and construct a network diagram of “core food–medicine homologous TCMs–active compounds–human-origin vascular aging targets” (Figure 4). The network graph includes 61 nodes and 478 edges. The red nodes represent top ten core “food–medicine homologous TCMs,” the purple nodes represent active compounds, and the green nodes represent human-origin vascular aging targets. The top ten core TCMs include 27 active compounds corresponding to 24 human-origin vascular aging targets. The top ten core “food–medicine homologous TCMs” may delay human-origin vascular aging by acting on targets such as TNF, peroxisome proliferator-activated receptor gamma (PPARγ), matrix metalloproteinase-1 (MMP-1), and IL-6. Key active compounds were identified based on the network topology, including top-ranked active compounds such as MOL000098 (Quercetin) (Table 2).

### 2.5. Results of Pathway Enrichment

**Figure 5:**
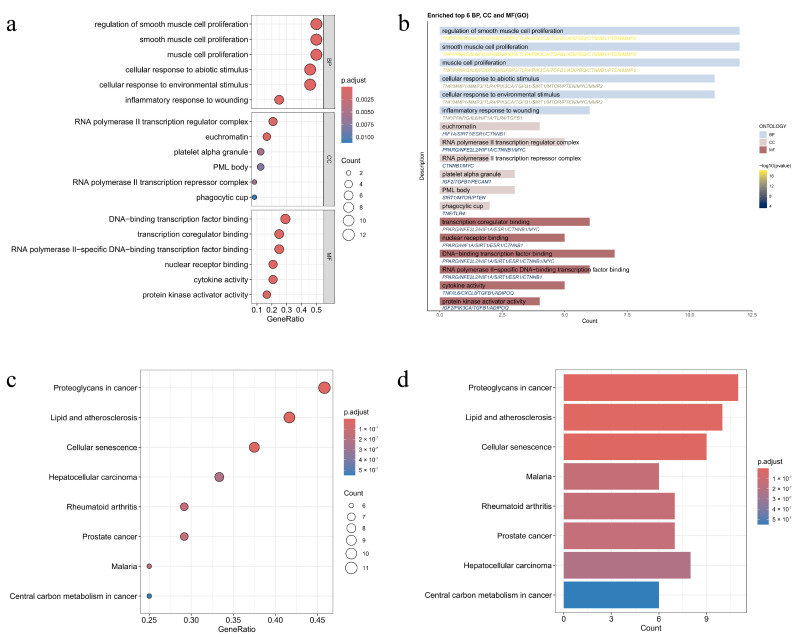
GO and KEGG enrichment analysis results (**a**) GO analysis bubble chart; (**b**) GO analysis bar chart; (**c**) KEGG analysis bubble chart; and (**d**) KEGG analysis bar chart.

Twenty-four human-origin vascular aging targets in the sub-network were enriched by the R Studio 4.4.0 package “Bioconductor.” Based on the screening criteria, *p*-value cutoff ≤ 0.05 and q-value cutoff ≤ 0.05, 1753 biological processes (BP), 20 cellular components (CC), and 54 molecular functions (MF) were obtained by GO analysis (Figure 5a,b). These targets are mainly involved in BP, such as the regulation of smooth muscle cell proliferation (GO:0048660), smooth muscle cell proliferation (GO:0048659), and muscle cell proliferation (GO:0033002), suggesting the importance of the above biological process in the delay of human-origin vascular aging by top ten core “food–medicine homologous TCMs.” CC analyses indicated that the RNA polymerase II transcription regulator complex (GO:0090575) and euchromatin (GO:0000791) regulate the intervention of human-origin vascular aging. MF analyses showed that the above targets may act through transcription coregulator binding (GO:0001221) and DNA-binding transcription factor binding (GO:0140297).

KEGG enrichment analysis revealed 117 pathways, suggesting that the core targets were mainly enriched in the pathways of proteoglycans in cancer (hsa05205), lipid and atherosclerosis (hsa05417), and cellular senescence (hsa04218) (Figure 5c,d). It is hypothesized that the top ten “food–medicine homologous TCMs” delaying human-origin vascular aging may be related to pathways such as proteoglycans in cancer (hsa05205). These pathways may intersect with vascular aging mechanisms via extracellular matrix (ECM) remodeling and cell proliferation regulation.

### 2.6. MMP-1, CXCL8, and PECAM1 as Core Targets

**Figure 6:**
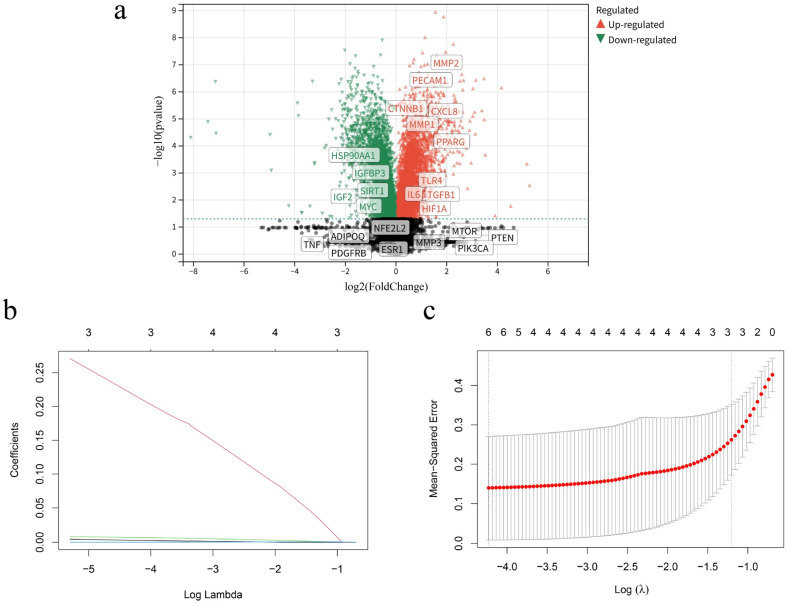
Identification of core targets. (**a**) Volcano diagram and target validation; (**b**) regression path diagram; and (**c**) cross-validation result diagram.

The 24 human-origin vascular aging targets identified above were validated against differentially expressed genes (logFC = 1 and *p* ≤ 0.05) in the GSE155680 dataset. The results showed that it contained 10 upregulated genes: *MMP-1*, *matrix metalloproteinase-2 (MMP-2)*, *platelet endothelial cell adhesion molecule-1 (PECAM1)*, *CTNNβ1*, *interleukin-8 (CXCL8)*, *PPARγ*, *toll-like receptor 4 (TLR4)*, *IL-6*, *TGFβ1*, and *hypoxia-inducible factor-1α (HIF1α)*. It also contained five downregulated genes: *heat shock protein 90 alpha family class A member 1 (HSP90αA1)*, *insulin-like growth factor 2 (IGF2)*, *insulin-like growth factor binding protein 3 (IGFBP3)*, *silent mating type information regulation 2 homolog 1 (SIRT1)*, and *myelocytomatosis oncogene (MYC)*. Nine targets were not differential genes: *TNF*, *platelet-derived growth factor receptor beta (PDGFRβ)*, *adiponectin (ADIPOQ)*, *estrogen receptor 1 (ESR1)*, *nuclear factor erythroid 2-related factor 2 (NFE2L2)*, *matrix metalloproteinase-3 (MMP-3)*, *mammalian target of rapamycin (mTOR)*, *phosphatidylinositol-4,5-bisphosphate 3-kinase catalytic subunit alpha (PIK3Cα)*, and *phosphate* and *tension homology deleted on chromosome ten (PTEN)* (Figure 6). The specific data for differentially expressed genes can be found in Supplementary Table S3. Fifteen human-origin vascular aging targets belonging to differential genes were analyzed by LASSO machine learning to screen the core targets: *MMP-1*, *CXCL8*, and *PECAM1*. The effect of *CXCL8* was the most significant (Figure 6b,c, Table 3).

### 2.7. MMP-1-β-Carotene Complexes with Optimal Binding Activity

**Figure 7:**
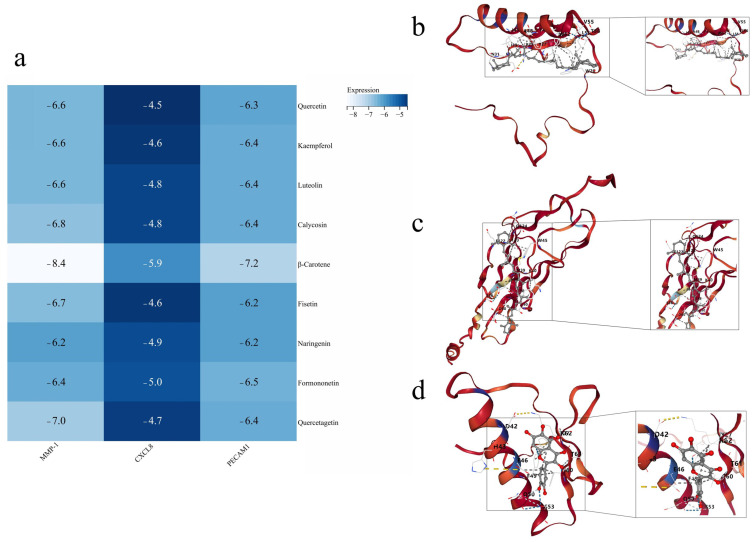
Molecular docking results. (**a**) Heat map display of molecular docking results; (**b**) optimal binding activity docking mode of MMP-1-β-Carotene complex; (**c**) optimal binding activity docking mode of PECAM1-β-Carotene complex; and (**d**) optimal binding activity docking mode of MMP-1-Quercetin complex.

To evaluate the plausibility of binding human-origin vascular aging targets to active compounds, molecular docking of core targets with important active compounds was performed. In the network of “core food–medicine homologous TCMs–active compounds–human-origin vascular aging targets,” including three core targets as receptor proteins and nine main active compounds as ligands, 27 molecular dockings were carried out. The results are displayed in the form of a heat map (Figure 7a). It is generally accepted that the smaller the binding energy of the receptor and ligand, the higher the binding activity. All 3 human-origin vascular aging core targets have good binding activity to the main active compounds. Among them, β-Carotene showed the highest binding activity to MMP-1, with a binding energy of −8.4 kcal/mol, followed by the PECAM1-β-Carotene complexes and MMP-1-Quercetagetin complexes. Figure 7b–d demonstrates the optimal binding activity docking pattern of some of the human-origin vascular aging core targets with the main active compounds.

### 2.8. MMP-1 Binds Well to β-Carotene

**Figure 8:**
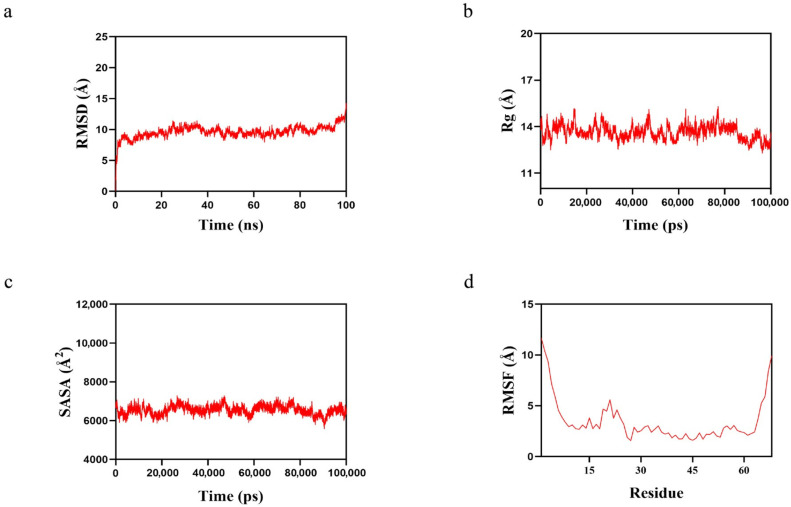
Molecular dynamics simulation results of the MMP-1-β-Carotene complex. (**a**) RMSD value over time; (**b**) Rg value over time; (**c**) SASA value over time; and (**d**) RMSF value.

According to the root mean square deviation (RMSD) results (Figure 8a), the MMP-1-β-Carotene complex system reached equilibrium after 5 ns and finally fluctuated up and down at 13.3 Å, showing high stability. According to the results of the radius of gyration (Rg) (Figure 8b), the MMP-1-β-Carotene complex system showed up-and-down fluctuations during the movement, indicating that the complex underwent conformational changes during the movement. According to the solvent accessible surface area (SASA) results (Figure 8c), the MMP-1-β-Carotene complex system showed slight fluctuations, demonstrating that binding small molecules affects the binding microenvironment and leads to some degree of SASA changes. Finally, according to the root mean square fluctuation (RMSF) results (Figure 8d), the complexes showed relatively low RMSF values (mostly below 3 Å), indicating low flexibility and high stability. Therefore, the MMP-1-β-Carotene complex system is more stable during exercise, and MMP-1 binds well to β-Carotene.

## 3. Discussion

Vascular aging is defined as degenerative changes in vascular structure and function that occur with age [10]. It has been suggested [3] that vascular aging is an important structural basis for the aging of various organs and systems in the human body and is also a common mechanism for the development of various aging-related chronic diseases. Moreover, simple vascular aging without risk factors can significantly elevate the risk of developing cardiovascular and cerebrovascular diseases. Given that cardiovascular diseases rank first in incidence among age-related conditions, this underscores the central role of vascular aging [11]. With the gradual aging of the population, the prevention and treatment of aging-related diseases has become a key issue that needs to be addressed urgently, and the delay of vascular aging is an important measure to reduce the occurrence and development of aging-related diseases. Therefore, it is urgent to shift the focus of prevention and treatment of vascular aging forward and to explore effective measures and mechanisms to delay vascular aging, which is of great significance in advancing the health and improving the quality of life of the elderly.

In recent years, TCMs have developed a unique approach and theoretical system for delaying vascular aging. In particular, “food–medicine homologous TCMs” have been proven to have the effect of delaying vascular aging. Epicatechin is present in “food–medicine homologous TCMs” (e.g., Shanzha) and has recently been reported [12] to have the ability to regulate the cyclic GMP-AMP synthase (cGAS)-stimulator of interferon genes (STING)-interferon regulatory factor 3 (IRF3) signaling pathway to inhibit vascular aging of vascular smooth muscle cells (VSMCs) in a high-glucose environment. In addition, Huangjing (*Polygonati rhizoma*) [13] delayed vascular aging in naturally aging rats, which was closely related to the inhibition of the activation of the ataxia telangiectasia and rad3-related protein (ATR)/checkpoint kinase 1 (Chk1) pathway: a key link in cellular aging. Although studies have shown that most of the “food–medicine homologous TCMs” have the effect of delaying vascular aging, the most critical “food–medicine homologous TCMs” have not yet been screened, and their potential molecular mechanisms still need to be investigated in greater depth.

In this study, we used a reverse network pharmacology approach to screen out the top ten core “food–medicine homologous TCMs” for delaying human-origin vascular aging: Xi Honghua (*Crocus sativus* L.), Gan Cao (*Glycyrrhiza uralensis Fisch.*), Ju Hua (*Chrysanthemum morifolium Ramat.*), Huang Qi (*Astragalus membranaceus* (*Fisch.*) *Bunge*), Huai Hua (*Sophora japonica* L.), Sha Ji (*Hippophae rhamnoides* L.), Ma Chixian (*Portulaca oleracea* L.), Jin Yinhua (*Lonicera japonica Thunb.*), Dai Daihua (*Citrus aurantium* L. *var. amara Engl.*), and Sang Ye (*Morus alba* L.). Previous studies have shown that the above ten core TCMs can delay human-origin vascular aging by slowing down oxidative stress, alleviating inflammation, regulating angiogenesis, and inhibiting the migration and proliferation of VSMCs. Oxidative stress is one of the main mechanisms causing vascular aging [14]. It has been shown [15] that Xi Honghua (*Crocus sativus* L.) can increase superoxide dismutase (SOD) and glutathione peroxidase (GSH-Px) activities, decrease malondialdehyde (MDA) levels, and inhibit lipid peroxidation in the serum of a D-galactose-induced rat-aging model. Hydrogen-protective chrysanthemum aqueous extracts [16] have recently been reported to prevent impairments in nitric oxide (NO) production, endothelial nitric oxide synthase (eNOS) protein levels, oxidative stress, and mitochondrial dysfunction, and to prevent palmitate-induced endothelial dysfunction through the maintenance of redox homeostasis. In addition, quercetin-3-methyl ether [17], a major constituent of Huai Hua (*Sophora japonica* L.), could alleviate endothelial damage due to oxidative stress in ECs by activating the phosphatidylinositol 3 kinase (PI3K)/Protein Kinase B (Akt) pathway. Sang Ye (*Morus alba* L.) extract [18], Ma Chixian (*Portulaca oleracea* L.) [19], Dai Daihua (*Citrus aurantium* L. *var. amara Engl.*) total flavonoids [20], and Jin Yinhua (*Lonicera japonica Thunb.*) polyphenols [21] were found to have a delayed oxidative stress effect. “Inflammaging” is a significant cellular change during vascular aging, and chronic low-grade inflammation is the most important pathological process leading to vascular endothelial dysfunction [22]. It has been shown [23] that Xi Honghua (*Crocus sativus* L.) is a key step in inflammatory vascular injury by blocking nuclear factor-κB (NF-κB)/p65 signaling, inhibiting monocyte chemoattractant protein-1 (MCP-1) and interleukin-8 (IL-8) expression, and suppressing the adhesion and infiltration of immune cells into the inflamed endothelium. Recently, it has been reported [24] that isoliquiritigenin attenuates the expression of pro-inflammatory factors (MCP-1 and IL-6) in ECs and attenuates the inflammatory response of vascular ECs. Isorhamnetin [25], as the main component in Sha Ji (*Hippophae rhamnoides* L.), significantly downregulated the production of tumor necrosis factor-α (TNF-α), interleukin-1β (IL-1β), and IL-6, and its vasculoprotective effect may be related to the inhibition of mitogen-activated protein kinases (MAPKs) and NF-κB activation. In addition, high doses of aqueous extracts of Ju Hua (*Chrysanthemum morifolium Ramat.*) inhibited the thickening condition of the thoracic aortic mesentery and reduced C-reactive protein (CRP) levels [26]. Several studies have shown that organisms increase the proportion of vascular aging ECs in vascular tissue with age, leading to impaired angiogenic capacity. Two important carotenoids extracted from Xi Honghua (*Crocus sativus* L.), saffronin and saffronaxanthin, have been used as natural biomedicines to help improve suboptimal health due to abnormal angiogenesis. Among them, saffronin promotes angiogenesis by increasing cell viability in human umbilical vein endothelial cells (HUVECs) [27]. With vascular aging, the heterogeneity of VSMCs becomes more and more obvious, showing high proliferation, migration, and secretory changes [28]. Recently, it has been shown [29] that isoliquiritigenin can inhibit the regulatory process of VSMCs proliferation and migration by mediating the growth factor receptor-bound protein 2 (GRB2)-Extracellular signal-regulated kinase 1/2 (ERK1/2) signaling pathway, which has a vasculoprotective effect.

According to the sub-network analysis of core “food–medicine homologous TCMs”, the top ten TCMs may delay human-origin vascular aging through their effects on 24 targets, such as TNF, PPARγ, MMP-1, and IL-6. The targets are divided into four main categories. The first category is immune and inflammatory response proteins, such as TNF [30], PPARγ [31], MMPs (MMP-1-MMP-3) [32,33,34], IL-6 [35], CXCL8 [36], TLR4 [37], TGFβ1 [38], and PECAM1 [39]. The second category is anti-oxidative stress and anti-inflammatory response proteins, such as NFE2L2 [40], ADIPOQ [41], and CTNNβ1 [42]. The third category is cell growth, proliferation, and apoptosis regulatory proteins, such as HIF1α [43], PDGFRβ [44], PIK3Cα [45], ESR1 [46], mTOR [47], and MYC [48]. The fourth category is metabolism regulatory proteins, such as IGF2 [49], IGFBP3 [50], and SIRT1 [51]. In addition, cellular homeostasis maintenance proteins (HSP90αA1) [52] and tumor suppressor proteins (PTEN) [53] are also included.

The present study predicts that the top ten core “food–medicine homologous TCMs” may exert a potential effect in delaying human-origin vascular aging by modulating the interleukin-17 (IL-17) signaling pathway, the advanced glycation end products (AGEs) receptor for advanced glycation end products (RAGE) signaling pathway, and the PI3K/Akt signaling pathway. For specific details regarding the ten major signal pathways associated with the top ten core “food–medicine homologous TCMs,” please refer to Supplementary Table S4. It has now been shown [54,55,56] that the IL-17 signaling pathway may be involved in inducing vascular aging by inducing inflammatory responses and oxidative stress processes associated with ECs aging. It has been demonstrated in existing studies [57,58] that the AGEs-RAGE signaling pathway can mediate pro-inflammatory effects and induce oxidative stress in cells such as ECs, VSMCs, vascular wall stroma, and mononuclear phagocytes, disrupting normal physiological functions and morphological structures of vascular ECs and ultimately leading to vascular aging. The AGEs-RAGE signaling pathway also leads to the proliferation of vascular wall stroma, which disrupts the structure of the vascular wall and induces the onset of vascular aging [59]. The PI3K/Akt signaling pathway was found to be closely related to vascular aging. Activation of the PI3K/Akt signaling pathway can alleviate intravascular metabolic disorders, reduce the expression of inflammatory factors and oxidative stress damage, and indirectly ameliorate vascular inflammatory damage and delay vascular aging [60,61,62]. The PI3K/Akt can also participate in intracellular signaling activation and regulate various cell cycle processes by regulating the tumor protein 53 (p53)/cyclin-dependent kinase inhibitor 1A (p21) (a key gene in the induction of ECs apoptosis) and other pathways, thereby exerting a role in regulating ECs senescence [63,64].

To screen the core targets, 24 target genes were analyzed by differential gene validation and LASSO machine learning, to screen the core targets-MMP-1, CXCL8, and PECAM1. An abnormal increase in MMP-1 disrupts the balance between MMP-1 and tissue inhibitors of metalloproteinase-1 (TIMP-1), and the collagenolysis effect is enhanced, destroying the integrity of the tissue structure of the vessel wall and thus damaging the blood vessels [65]. In addition to this, increased MMP-1 expression causes neutrophils to release TNF-α and ROS on the one hand, and interacts with VSMCs to release IL-8 and MMP-1 on the other hand, which increases the cascade-effect style of the local inflammatory response and exacerbates vascular endothelial injury [65]. CXCL8, as an important inflammatory transmitter, mediates the aggregation of neutrophils and other inflammatory cells towards the site of inflammation, which in turn triggers a series of inflammatory responses [66]. PECAM1 is associated with inflammation and cell migration. PECAM1 may induce the early stages of inflammation and is also a major component in causing leukocyte extravasation, and its expression promotes leukocyte migration [39]. It has been reported [39] that PECAM1 is involved in leukocyte migration across endothelial cells. It can be seen that inhibiting inflammatory responses is most important for the top ten core “food–medicine homologous TCMs” delaying human-origin vascular aging.

According to the analysis of the sub-network analysis of core “food–medicine homologous TCMs,” Quercetin, Kaempferol, Luteolin, Calycosin, β-Carotene, Fisetin, and Naringenin may be the main components to delay human-origin vascular aging. The molecular docking results showed that the above main components exhibited good binding properties with the key targets (MMP-1, CXCL8, and PECAM1); this provides preliminary support for the validity of the screening results. Among them, the MMP-1-β-Carotene complex had the lowest binding energy (−8.4 kcal/mol), indicating that among the key targets selected in this study, β-Carotene demonstrated a relatively superior binding affinity for MMP-1. The stability of the MMP-1-β-Carotene complex was further confirmed by molecular dynamics simulations, suggesting that MMP-1 may be a potential target for β-Carotene. Therefore, it is hypothesized that MMP-1 is the core target and that the top ten core “food–medicine homologous TCMs” may target MMP-1 to play a role in delaying human-origin vascular aging.

MMP-1 is the most widely expressed mesenchymal collagenase in the family of matrix metalloproteinases (MMPs) that degrades collagen types I, II, and III and plays a key role in the initial cleavage of the extracellular matrix [67,68]. MMP-1 is mainly secreted by cells [67,68,69,70] such as VSMCs, macrophages, fibroblasts, and ECs, and is expressed at low levels in healthy states and at significantly higher levels in pathological conditions [67,71]. Several lines of evidence [72,73,74,75,76,77] suggest that the source of elevated MMP-1 production is localized in the vascular system and is a key player in most vascular diseases. MMP-1 is closely associated with repair response after large arterial wall injury in human atherosclerotic lesions, endothelial thickening, and plaque rupture [74,75,76]. In clinical studies [77], increased levels of MMP-1 expression have been found to increase the incidence of myocardial infarction. Recent studies have confirmed that MMP-1 is by far the most significantly upregulated MMP during cellular senescence in vitro and is highly associated with cellular senescence and age-related diseases. It has been shown [78] that upregulation of MMP-1 expression is an early event in lithium-induced endothelial cell senescence. Previous studies [79] have reported that inflammatory cytokine markers (IL-1β, TNF-α, etc.) stimulate MMP-1 secretion from peri-inflammatory tissue matrices during aging. On the one hand, MMP-1 promotes the spread of inflammation by hydrolyzing matrix components surrounding inflammation, exacerbating local inflammatory symptoms, and inducing vascular endothelial dysfunction [80]. On the other hand, activation of MMP-1 can sever the connection between ECs and their endothelial matrix, prompting ECs to peel off from the endothelium and exacerbating endothelial injury [80]. Recent studies [81,82,83] have shown the presence of the MMP-1-protease activated receptor 1 (PAR1) signaling system in the vascular endothelium, which induces a pro-thrombotic, pro-inflammatory, and adhesive microenvironment that triggers vascular endothelial dysfunction. Endothelial dysfunction and vascular endothelial injury can exacerbate vascular aging. Angiotensin II (Ang II) is increased in aging arterial walls, such as those of rats and nonhuman primates, and particularly in the thickened lining of humans [84]. Available data [85] suggest that Ang II can stimulate human VSMCs to secrete activated MMP-1 through the NF-κB and activator protein-1 (AP-1) signaling pathways, triggering VSMCs migration and proliferation, which may exacerbate vascular aging. These findings further link MMP-1 expression to the process of vascular aging.

It should be further clarified that vascular aging is not solely mediated by MMP-1 alone. When considering MMP-1 as a core target for vascular aging in this study, the role of other MMPs family members in vascular aging must also be taken into account. Unlike MMP-1, which primarily degrades type I and III fibrillar collagen, MMP-2 and matrix metalloproteinase-9 (MMP-9), as members of the gelatinase subfamily, reside within the ECM and are capable of degrading numerous ECM components, including type IV, V, VII, and X collagen; elastin; laminin; fibronectin; and numerous other ECM components, such as proteoglycans [86]. They mediate both physiological vascular tissue remodeling and pathological injury processes. Research indicates that downregulation of MMP-2 expression inhibits the proliferation and migration of VSMCs, thereby suppressing vascular remodeling [87]. Although consistent with MMP-1’s effects on VSMCs, their regulatory mechanisms differ. MMP-1 primarily influences VSMCs by degrading fibrillar collagen [67,85]. MMP-2 primarily degrades gelatin and type IV collagen, leading to the transendothelial migration of VSMCs [87]. Furthermore, research indicates that the upregulation of MMP-9 accelerates the degradation of damaged aortic endothelium, exacerbating endothelial rupture and mesarterial lesions, and degrading the cellular extracellular matrix provides the necessary space for plaque development [88]. MMP-2/9 participates in the regulation of vascular remodeling in parallel with MMP-1. Therefore, it is reasonable to propose that MMP-2/9 may be involved in the regulatory role of MMP-1 in vascular aging. Moreover, MMPs’ activity is not solely determined by their expression levels but also depends on the tissue inhibitors of metalloproteinases (TIMPs) [89]. This equilibrium system is crucial for maintaining vascular homeostasis. Research indicates [89] that tissue inhibitors of metalloproteinase-3 (TIMP-3) are the sole members of the TIMPs family that are exclusively present within the ECM and that TIMP-3 inhibits the vast majority of MMPs, including MMP-1. Research has confirmed [90] that the dynamic equilibrium between MMP-1 and TIMP-3 is a key factor in maintaining vascular endothelial homeostasis. An imbalance in their ratio disrupts the synthesis and degradation of syndecan-1, thereby compromising the integrity of the vascular endothelial barrier and ultimately inducing endothelial dysfunction. Research indicates [91] that overexpression of TIMP-3 in rat vascular smooth muscle cells promotes apoptosis, yet this effect is independent of its inhibitory action on MMPs. It is suggested that TIMP-3 may participate in the regulation of vascular cells via an MMP-independent pathway. This study predicts MMP-1 as a core target in human vascular aging, and its mechanism for delaying vascular aging requires further investigation in conjunction with MMPs and TIMPs.

Pathway enrichment analysis of the targets that directly interact with MMP-1 revealed significant associations with signaling pathways related to lipid and atherosclerosis, rheumatoid arthritis, and the IL-17 signaling pathway. For specific details regarding MMP-1-related signaling pathways, please refer to Supplementary Table S5. The results of this study suggest that the IL-17 signaling pathway is an important correlative feature of the top ten core “food–medicine homologous TCMs” in targeting MMP-1 and potentially contributing to the attenuation of human-origin vascular aging. It has been demonstrated [92,93,94] that the IL-17 signaling pathway may be involved in inducing vascular aging by inducing inflammatory responses and oxidative stress processes associated with ECs aging. IL-17, as a pro-inflammatory factor, binds to the receptor interleukin-17 receptor (IL-17R), and through the signal transduction complex IL-17R-nuclear factor kappa B activation agent 1 (Act1)-TNF receptor-associated factor 6 (TRAF6), it can further activate the nuclear factor NF-κB and MAPK pathways to regulate cell differentiation/growth, cell adhesion, and apoptosis, as well as inflammatory responses [95,96]. It has been reported [97,98] that the NF-kB and MAPK pathways promote the expression of various inflammatory genes, such as granulocyte-macrophage colony-stimulating factor (GM-CSF), MCP-1, and cysteine-x-cysteine chemokines (CXC), which mediate inflammatory cell infiltration and tissue damage. The production of inflammatory factors contributes to increased production of MMP-1 in vascular ECs and VSMCs, exacerbating the inflammatory state of the vasculature, contributing to VSMCs migration and proliferation, and exacerbating vascular aging [99]. More importantly, IL-17 is secreted in large quantities by T helper cell 17 (Th17), a subset of CD4+ T cells [100]. In immunosenescence, the senescent Treg cell population is characterized by phenotypic switching into T helper 1 cell (Th1), Th17, and follicular helper T cell (Tfh) subtypes with age [94]. Accordingly, we hypothesize that immunosenescence may be a prerequisite for vascular aging. The top ten core “food–medicine homologous TCMs” may be able to delay human-origin vascular aging by targeting immunosenescence. This could be a new idea to delay human-origin vascular aging.

This study has the following limitations. Firstly, it relies heavily on computational simulation methods, such as machine learning algorithms and molecular docking. Consequently, conclusions regarding targets and mechanisms of human-origin vascular aging remain speculative, lacking support from in vitro or in vivo experimental data. Secondly, all data analyzed originated from databases, including NCBI, the Aging Atlas database, and GEO. Potential database bias may compromise the accuracy of results. Thirdly, the study utilized only the NCBI and Aging Atlas databases to screen for human-origin vascular aging targets, omitting analysis of additional databases and potentially overlooking certain candidate targets. Fourthly, existing data sources encompass both in vivo and in vitro studies, lacking clinical data support. Fifthly, the study employed the Traditional Chinese Medicine System Pharmacology Database (TCMSP) platform to screen the chemical composition of “food–medicine homologous TCMs”. However, the chemical composition of TCMs is complex, with many chemical compositions yet to be fully characterized and annotated. Furthermore, the prediction of OB/DL values carries inherent inaccuracies, which may impact the scope of the screening. Sixth, the LASSO machine learning employed for core target screening may overlook low-expression or unannotated genes, due to its variable compression preferences.

## 4. Materials and Methods

### 4.1. Screening of Human-Origin Vascular Aging Genes

The NCBI gene databases integrate gene-specific information from multiple data sources and are used for research in genetics, molecular biology, and bioinformatics. The Aging Atlas database integrates aging-related datasets from the conventional transcriptome, single-cell transcriptome, epigenome, proteome, and pharmacogenome levels and, for the first time, achieves the inclusion and integration of multi-omics data related to aging at different levels. In this study, human-origin VAGs were first collected from the NCBI gene database (www.ncbi.nlm.nih.gov/gene, accessed on 17 April 2025). Subsequently, human-origin AAGs were obtained from the Aging Atlas database (https://ngdc.cncb.ac.cn/aging/index, accessed on 17 April 2025). Human-origin VAGs were crossed with human-origin AAGs, and human-origin vascular aging-associated genes were ultimately selected as the target dataset in this study.

### 4.2. PPI Network Construction and Core Target Identification

To screen for core targets of human-origin vascular aging, human-origin vascular aging-associated genes were imported as targets into the STRING database (https://cn.string-db.org/, accessed on 21 April 2025). The species was restricted to “Homo sapiens,” the minimum interaction threshold was set to “high confidence ≥ 0.7,” and the free target was hidden to generate a PPI network diagram. The network was visualized with the assistance of the software Cytoscape 3.8.0. We performed a network topology analysis using the CytoNCA plug-in and designated targets that exceeded the average node degree value as core targets.

### 4.3. Active Compound and TCMs Screening

The protein names of the human-origin vascular aging core targets were entered into the TCMSP (https://www.tcmsp-e.com/, accessed on 23 April 2025) to search for relevant chemical components. The active compounds were collected with the constraints of OB ≥ 30% [101], DL index ≥ 0.18 [102], and HL ≥ 4 [8,103]. The active compounds obtained were entered into the High-throughput Experiment- and Reference-guided database of the TCM (HERB) (http://herb.ac.cn/, accessed on 23 April 2025) and TCMSP databases to identify relevant TCMs [8]. Subsequently, TCMs not belonging to the catalog of “food–medicine homologous TCMs” published by the National Health and Health Commission were excluded. Cytoscape 3.8.0 was used to construct a network map of “human-origin vascular aging core targets–active compounds–food–medicine homologous TCMs”. The Cytoscape 3.8.0 topology tool was used to calculate the node degree values to screen core TCMs from “food–medicine homologous TCMs”.

### 4.4. Pathway Enrichment Analysis

Cytoscape 3.8.0 software was used to extract the sub-networks associated with the core TCMs from “food–medicine homologous TCMs” and construct a network diagram of “core food–medicine homologous TCMs–active compounds–human-origin vascular aging targets”. Using the R Studio 4.4.0 package “clusterProfiler (4.14.6)”, with pvalueCutoff ≤ 0.05 and qvalueCutoff ≤ 0.05. GO and KEGG enrichment analyses were then performed on the human-origin vascular aging targets that had previously been screened. GO analysis includes BP, MF, and CC, and obtains the pathways involved by the targets in the core sub-network, finally visualizing them.

### 4.5. Target Validation and Core Target Screening

HUVECs senescence gene expression data were obtained from the GEO database (https://www.ncbi.nlm.nih.gov/geo/, accessed on 30 April 2025). First, datasets using HUVECs as the study subject were prioritized. Further screening focused on datasets employing natural aging induction methods to exclude interference from exogenous inducers. Finally, based on the dataset abstract information and grouping design, pure datasets exclusively investigating the HUVEC senescence process were selected. The GSE155680 dataset file was downloaded from the GPL18460 platform. The differential genes in this dataset were obtained via differential gene analysis, using the “limma (3.62.2)” package in R 4.4.0 with logFC = 1 and *p* ≤ 0.05. After target validation, the targets belonging to the differential genes were analyzed by the LASSO machine learning algorithm, using the “glmnet (4.1-8)” package in R 4.4.0 to screen the core targets.

### 4.6. Molecular Docking and Dynamics Simulation

In the core “food–medicine homologous TCMs” sub-network, the above-screened human-origin vascular aging core targets were used as receptors, and the active compounds with top-ranked degree values were used as ligands. The receptor protein structures were downloaded from the UniProt database (https://www.uniprot.org/, accessed on 5 May 2025) and the ligand structures from the PubChem database (https://pubchem.ncbi.nlm.nih.gov/, accessed on 5 May 2025). Protein and ligand molecules were pretreated using PyMOL 3.1.0 software. Molecular docking was performed in CB-Dock2 (https://cadd.labshare.cn/cb-dock2/php/index.php, accessed on 10 May 2025) to predict the binding energy of the receptor to the ligand, select the lowest binding energy as the best conformation, and visualize the docking results [104]. The receptor and ligand with the best conformation were subjected to molecular dynamics simulations, using Gromacs 2022 [105,106]. During the simulations, the g-rmsd, g-rmsf, g-Rg, and g-sasa tools were used to calculate the RMSD, RMSF, Rg, and SASA, respectively.

## 5. Conclusions

In conclusion, this study using inverse network pharmacology, bioinformatics, machine learning, molecular docking, and molecular dynamics simulation, systematically screened out the top ten core “food–medicine homologous TCMs” with the greatest potential to delay vascular aging for the first time. The findings of this study suggest that β-Carotene within these core “food–medicine homologous TCMs” may represent a potential active component targeting MMP-1, with its action potentially linked to the IL-17 signaling pathway. These ten top “food–medicine homologous TCMs” may offer potential candidate directions for developing functional products that delay vascular aging while simultaneously providing computationally predicted mechanistic insights and a scientific rationale for novel therapies targeting vascular aging. The present study highlights the new hypothesis that immunosenescence (Th17/IL-17) is involved in vascular aging, suggesting that the top ten core “food–medicine homologous TCMs” may delay vascular aging by modulating immune cells. This may provide a new perspective for the development of targets and drugs to delay vascular aging.

## Figures and Tables

**Table 1:** Specific information on key targets of human-origin vascular aging.

Gene Symbol	Protein Name	Degree
IL-6	Interleukin-6	58.0
TNF	Tumor necrosis factor	54.0
IGF1	Insulin-like growth factor 1	46.0
CTNNβ1	β-Catenin	44.0
TGFβ1	Transforming growth factor β1	44.0
HIF1α	Hypoxia-inducible factor-1α	36.0
HSP90αA1	Heat shock protein 90 alpha family class A member 1	36.0
PTEN	Phosphate and tension homology deleted on chromosome ten	34.0
PIK3Cα	Phosphatidylinositol-4,5-bisphosphate 3-kinase catalytic subunit alpha	34.0
MMP-2	Matrix metalloproteinase-2	34.0
MYC	Myelocytomatosis oncogene	32.0
SIRT1	Silent mating type information regulation 2 homolog 1	32.0
CXCL12	Stromal cell-derived factor-1	30.0
PPARγ	Peroxisome proliferator-activated receptor gamma	30.0
ESR1	Estrogen receptor 1	28.0
IGF2	Insulin-like growth factor 2	26.0
mTOR	Mammalian target of rapamycin	26.0
IGF1R	Insulin-like growth factor 1 receptor	26.0
CXCL8	Interleukin-8	26.0
TLR4	Toll-like receptor 4	24.0
PECAM1	Platelet endothelial cell adhesion molecule-1	22.0
PDGFRα	Platelet-derived growth factor receptor α	22.0
FLT1	Vascular endothelial growth factor receptor 1	22.0
APOE	Apolipoprotein E	22.0
IGFBP3	Insulin-like growth factor-binding protein 3	18.0
PDGFRβ	Platelet-derived growth factor receptor beta	18.0
MMP-3	Matrix metalloproteinase-3	18.0
α2M	α-2-macroglobulin	18.0
MMP-1	Matrix metalloproteinase-1	16.0
APP	Gamma-secretase C-terminal fragment 50	16.0
NFE2L2	Nuclear factor erythroid 2-related factor 2	14.0
STK11	Serine/threonine kinase 11	12.0
FGFR1	Fibroblast growth factor receptor 1	12.0
TNFSF11	Tumor necrosis factor ligand superfamily member 11	12.0
ADIPOQ	Adiponectin	12.0
HMGB1	High mobility group box 1 protein	10.0
CDC42	Cell division control protein 42 homolog	10.0
BMP2	Bone morphogenetic protein 2	10.0
MAPT	Microtubule-associated protein tau	10.0

**Table 2:** Information on some active compounds.

Mol ID	Molecule Name	Oral Bioavailability (OB) (%)	Drug Likeness (DL)	Half Life (HL)	Degree
MOL000098	Quercetin	46.43	0.28	14.4	198.0
MOL000422	Kaempferol	41.88	0.24	14.74	66.0
MOL000006	Luteolin	36.16	0.25	15.94	40.0
MOL000417	Calycosin	47.75	0.24	17.1	24.0
MOL002773	β-Carotene	37.18	0.58	4.36	14.0
MOL013179	Fisetin	52.6	0.24	15.06	14.0
MOL004328	Naringenin	59.29	0.21	16.98	12.0
MOL000392	Formononetin	69.67	0.21	17.04	12.0
MOL002721	Quercetagetin	45.01	0.31	13.82	12.0

**Table 3:** Core gene coefficients.

Serial Number	Gene	Coef
1	(Intercept)	−11.8280998267896
2	MMP-1	0.00277684619713685
3	CXCL8	0.215024075604593
4	PECAM1	0.00704157295786899

## Data Availability

The original contributions presented in this study are included in the article/Supplementary Material. Further inquiries can be directed to the corresponding author.

## Supplementary Materials

The following supporting information can be downloaded at: https://www.mdpi.com/article/10.3390/ph19010131/s1, Table S1: Human-origin vascular aging-associated genes; Table S2: The specific degree values for the “food-medicine homologous TCMs”; Table S3: The specific data for differentially expressed genes; Table S4: The ten major signal pathways associated with the top ten core “food-medicine homologous TCMs”; Table S5: Regarding MMP-1-related signaling pathways.

## Abbreviations

The following abbreviations are used in this manuscript:

AAGs	Aging-associated genes
Act1	Nuclear factor kappa B activation agent 1
ADIPOQ	Adiponectin
AGEs	Advanced glycation end products
Akt	Protein Kinase B
Ang II	Angiotensin II
AP-1	Activator protein-1
Atg7	Autophagy-related protein 7
ATR	Ataxia telangiectasia and rad3-related protein
BP	Biological process
CC	Cellular component
cGAS	Cyclic GMP-AMP synthase
Chk1	Checkpoint kinase 1
CRP	C-reactive protein
CTNNβ1	β-Catenin
CXC	Cysteine-x-cysteine chemokines
DL	Drug likeness
ECs	Endothelial cells
ECM	Extracellular matrix
eNOS	Endothelial nitric oxide synthase
EPCs	Endothelial progenitor cells
ERK1/2	Extracellular signal-regulated kinase 1/2
ESR1	Estrogen receptor 1
GEO	Gene Expression Omnibus
GM-CSF	Granulocyte-macrophage colony stimulating factor
GO	Gene Ontology
GRB2	Growth factor receptor-bound protein 2
GSH-Px	Glutathione peroxidase
HERB	High-throughput Experiment- and Reference-guided database of TCM
HIF1α	Hypoxia-inducible factor-1α
HL	Half life
HSP90αA1	Heat shock protein 90 alpha family class A member 1
HUVEC	Human umbilical vein endothelial cell
IGF1	Insulin-like growth factor 1
IGF2	Insulin-like growth factor 2
IGFBP3	Insulin-like growth factor binding protein 3
IL-1β	Interleukin-1β
IL-6	Interleukin-6
IL-8/CXCL8	Interleukin-8
IL-17	Interleukin-17
IL-17R	Interleukin-17 receptor
IRF3	Interferon regulatory factor 3
KEGG	Kyoto Encyclopedia of Genes and Genomes
LASSO	Least absolute shrinkage and selection operator
LC3	Microtubule-associated protein 1 light chain 3
MAPK	Mitogen-activated protein kinase
MCP-1	Monocyte chemoattractant protein-1
MDA	Malondialdehyde
MF	Molecular function
MMPs	Matrix metalloproteinases
MMP-1	Matrix metalloproteinase-1
MMP-2	Matrix metalloproteinase-2
MMP-3	Matrix metalloproteinase-3
MMP-9	Matrix metalloproteinase-9
mTOR	Mammalian target of rapamycin
MYC	Myelocytomatosis oncogene
NCBI	National Center for Biotechnology Information
NFE2L2	Nuclear factor erythroid 2-related factor 2
NF-κB	Nuclear factor-κB
NO	Nitric oxide
OB	Oral bioavailability
PAR1	Protease activated receptor 1
PDGFRβ	Platelet-derived growth factor receptor beta
PECAM1	Platelet endothelial cell adhesion molecule-1
PIK3Cα	Phosphatidylinositol-4,5-bisphosphate 3-kinase catalytic subunit alpha
PI3K	Phosphatidylinositol 3 kinase
PPI	Protein–protein interaction
PPARγ	Peroxisome proliferator-activated receptor gamma
PTEN	Phosphate and tension homology deleted on chromosome ten
p21	Cyclin-dependent kinase inhibitor 1A
p53	Tumor protein 53
p62	Sequestosome-1
RAGE	Receptor for advanced glycation end products
Rg	Radius of gyration
RMSD	Root mean square deviation
RMSF	Root mean square fluctuation
ROS	Reactive oxygen species
SASA	Solvent accessible surface area
SIRT1	Silent mating type information regulation 2 homolog 1
SOD	Superoxide dismutase
STRING	Search Tool for the Retrieval of Interacting Genes/Proteins
STING	Stimulator of interferon genes
TCMs	Traditional Chinese Medicines
TCMSP	Traditional Chinese Medicine System Pharmacology Database
Tfh	Follicular helper T cell
TGFβ1	Transforming growth factor β1
Th1	T helper 1 cell
Th17	T helper cell 17
TIMPs	Tissue inhibitors of metalloproteinases
TIMP-1	Tissue inhibitors of metalloproteinase-1
TIMP-3	Tissue inhibitors of metalloproteinase-3
TLR4	Toll-like receptor 4
TNF	Tumor necrosis factor
TNF-α	Tumor necrosis factor-α
TRAF6	TNF receptor-associated factor 6
VAGs	Vascular-associated genes
VSMCs	Vascular smooth muscle cells

## References

[1] Partridge L., Deelen J., Slagboom P.E. (2018). Facing up to the global challenges of ageing. Nature.

[2] Costantino S., Cosentino F. (2015). Ageing, metabolism and cardiovascular disease. J. Physiol..

[3] Zhang C., Tao J. (2018). Expert consensus on clinical assessment and intervention of vascular aging in China. Aging Med..

[4] Qi Z., Wu D., Zhao J., Hui J., Zhang Y., Li T., Xu F. (2024). Research progress and thinking on pharmacological effect of traditional Chinese medicine in treatment of cardiovascular aging. Chin. Tradit. Herb. Drugs.

[5] Yang M., Liang Z., Ge L., Lan W. (2022). Study on History of Development and Laws of Herbal Property About Homology of Medicine and Food. Chin. J. Ethnomed. Ethnopharm..

[6] Zai Q., Qin Z., Ye L. (2016). Effect of Rhizoma Polygonati on the Function of Endothelial Progenitor Cells and Telomerase Activity in Nature Senescent Rats. Chin. J. Integr. Tradit. West. Med..

[7] Liu P., Zhao H., Luo Y. (2017). Anti-Aging Implications of *Astragalus membranaceus* (Huangqi): A Well-Known Chinese Tonic. Aging Dis..

[8] Luo T., Zhao Z., Wu M., Ren X., Xu Z., Li L., Yi Y., Wang H., Wang L. (2024). Network pharmacology screening, in vitro and in vivo evaluation of antianxiety and antidepressant drug-food analogue. Phytomed. Int. J. Phytother. Phytopharm..

[9] Priyamvada P., Ashok G., Joshi T., Anbarasu S., Anbarasu A., Ramaiah S. (2025). Unravelling the molecular mechanistic pathway underlying the anticancer effects of kaempferol in colorectal cancer: A reverse pharmacology network approach. Mol. Divers..

[10] LaRocca T.J., Martens C.R., Seals D.R. (2016). Nutrition and other lifestyle influences on arterial aging. Ageing Res. Rev..

[11] Jiang P., Li J. (2014). Vascular Aging and The Corresponding Mechanisms. Prog. Biochem. Biophys..

[12] Zhang L., Lin H., Chen Z., Zhang H., Tong J., Li Y. (2025). Epicatechin Reduces Diabetic Vascular Smooth Muscle Aging by Regulating the cGAS-STING-IRF3 Pathway. Guid. J. Tradit. Chin. Med. Pharm..

[13] Shi Y., Feng J., Liang M., Xiao Z., Qin Z. (2023). Effect of Polygonati Rhizoma Regulating ATR/Chk1 Pathway on Vascular Aging in Naturally Aged Rats. Tradit. Chin. Drug Res. Clin. Pharmacol..

[14] Puca A.A., Carrizzo A., Villa F., Ferrario A., Casaburo M., Maciąg A., Vecchione C. (2013). Vascular ageing: The role of oxidative stress. Int. J. Biochem. Cell Biol..

[15] Wang Q., Zhang L., Su H., Wan X. (2020). Effect of Saffron on Antioxidant Capacity of D-Galactose-Induced Aging Rat Model. Chin. Arch. Tradit. Chin. Med..

[16] Gao Y., Song O., Wang M., Guo X., Zhang G., Liu X., Liu J., Zhao L. (2023). Hydrogen Protection Boosts the Bioactivity of Chrysanthemum morifoliumExtract in Preventing Palmitate-Induced Endothelial Dysfunction by Restoring MFN2 and Alleviating Oxidative Stress in HAEC Cells. Antioxidants.

[17] Tseng H.-L., Li C.-J., Huang L.-H., Chen C.-Y., Tsai C.-H., Lin C.-N., Hsu H.-Y. (2012). Quercetin 3-O-methyl ether protects FL83B cells from copper induced oxidative stress through the PI3K/Akt and MAPK/Erk pathway. Toxicol. Appl. Pharmacol..

[18] Shibata Y., Kume N., Arai H., Hayashida K., Inui-Hayashida A., Minami M., Mukai E., Toyohara M., Harauma A., Murayama T. (2006). Mulberry leaf aqueous fractions inhibit TNF-alpha-induced nuclear factor kappaB (NF-kappaB) activation and lectin-like oxidized LDL receptor-1 (LOX-1) expression in vascular endothelial cells. Atherosclerosis.

[19] Miao L., Zhou C., Zhang H., Cheong M., Tan Y., Wang Y., Zhang X., Yu H., Cheang W. (2023). *Portulaca oleracea* L. (Purslane) Extract Protects Endothelial Function by Reducing Endoplasmic Reticulum Stress and Oxidative Stress through AMPK Activation in Diabetic Obese Mice. Antioxidants.

[20] Shen C.-Y., Wang T.-X., Zhang X.-M., Jiang J.-G. (2017). Various Antioxidant Effects Were Attributed to Different Components in the Dried Blossoms of *Citrus aurantium* L. var. *amara* Engl. J. Agric. Food Chem..

[21] Zheng S.-L., Wang Y.-M., Chi C.-F., Wang B. (2024). Chemical Characterization of Honeysuckle Polyphenols and Their Alleviating Function on Ultraviolet B-Damaged HaCaT Cells by Modulating the Nrf2/NF-κB Signaling Pathways. Antioxidants.

[22] Aicher A., Zeiher A.M., Dimmeler S. (2005). Mobilizing endothelial progenitor cells. Hypertension.

[23] Song L., Kang C., Sun Y., Huang W., Liu W., Qian Z. (2016). Crocetin Inhibits Lipopolysaccharide-Induced Inflammatory Response in Human Umbilical Vein Endothelial Cells. Cell. Physiol. Biochem. Int. J. Exp. Cell. Physiol. Biochem. Pharmacol..

[24] Wang L., Zhu R., He C., Li H., Zhang Q., Cheung Y., Leung F., Wong W. (2024). Licorice Extract Isoliquiritigenin Protects Endothelial Function in Type 2 Diabetic Mice. Nutrients.

[25] Chi G., Zhong W., Liu Y., Lu G., Lü H., Wang D., Sun F. (2015). Isorhamnetin protects mice from lipopolysaccharide-induced acute lung injury via the inhibition of inflammatory responses. Inflamm. Res..

[26] Wang S., Zhu Q., Yin X., Wu S., Wang B., Nie J., Ye X., Liu D. (2023). Antihypertensive effect of Chrysanthemum morifolium extracts on hypertensive rats with liver prosperity and phlegm obstruction syndrome. Cent. South Pharm..

[27] Zhao C., Kam H., Chen Y., Gong G., Hoi M.P.-M., Skalicka-Woźniak K., Dias A.C.P., Lee S.M.-Y. (2021). Crocetin and Its Glycoside Crocin, Two Bioactive Constituents From *Crocus sativus* L. (Saffron), Differentially Inhibit Angiogenesis by Inhibiting Endothelial Cytoskeleton Organization and Cell Migration Through VEGFR2/SRC/FAK and VEGFR2/MEK/ERK Signaling Pathways. Front. Pharmacol..

[28] Lakatta E.G., Levy D. (2003). Arterial and cardiac aging: Major shareholders in cardiovascular disease enterprises: Part I: Aging arteries: A “set up” for vascular disease. Circulation.

[29] Qin L., Gao X., Gao L., Li Y., Zhao J. (2025). Isoliquiritigenin (ISL) inhibits proliferation and migration of vascular smooth muscle cells by regulating GRB2/ERK signaling. Chin. Pharmacol. Bull..

[30] Jang D.-I., Lee A.-H., Shin H.-Y., Song H.-R., Park J.-H., Kang T.-B., Lee S.-R., Yang S.-H. (2021). The Role of Tumor Necrosis Factor Alpha (TNF-α) in Autoimmune Disease and Current TNF-α Inhibitors in Therapeutics. Int. J. Mol. Sci..

[31] Lian M., Tao Y., Chen J., Shen X., Hou L., Cao S., Fang J. (2021). Variation of PPARG Expression in Chemotherapy-Sensitive Patients of Hypopharyngeal Squamous Cell Carcinoma. PPAR Res..

[32] Rodrı D., Morrison C., Overall C.M. (2009). Matrix metalloproteinases: What do they not do? New substrates and biological roles identified by murine models and proteomics. Biochim. Biophys. Acta.

[33] Wang X.L., Khalil R.A. (2017). Matrix Metalloproteinases, Vascular Remodeling, and Vascular Disease. Adv. Pharmacol..

[34] Sun S., Bay-Jensen A.-C., Karsdal M.A., Siebuhr A.S., Zheng Q., Maksymowych W.P., Christiansen T.G., Henriksen K. (2014). The active form of MMP-3 is a marker of synovial inflammation and cartilage turnover in inflammatory joint diseases. BMC Musculoskelet. Disord..

[35] Huang B., Lang X., Li X. (2022). The role of IL-6/JAK2/STAT3 signaling pathway in cancers. Front. Oncol..

[36] Zhu J., Song C., Cai T., Yi L., Zhang W., Zhong J., Shen M. (2022). The Relationship between Serum CXCL8 and ET-1 Expression Levels and Sepsis Complicated with Heart Failure. Cardiol. Res. Pract..

[37] Park B.S., Lee J.-O. (2013). Recognition of lipopolysaccharide pattern by TLR4 complexes. Exp. Mol. Med..

[38] Moreau J., Velegraki M., Bolyard C., Rosenblum M.D., Li Z. (2022). Transforming growth factor-β1 in regulatory T cell biology. Sci. Immunol..

[39] Dangerfield J., Larbi K.Y., Huang M.-T., Dewar A., Nourshargh S. (2002). PECAM-1 (CD31) homophilic interaction up-regulates alpha6beta1 on transmigrated neutrophils in vivo and plays a functional role in the ability of alpha6 integrins to mediate leukocyte migration through the perivascular basement membrane. J. Exp. Med..

[40] Peng H., Wang H., Xue P., Hou Y., Dong J., Zhou T., Qu W., Peng S., Li J., Carmichael P.L. (2015). Suppression of NRF2-ARE activity sensitizes chemotherapeutic agent-induced cytotoxicity in human acute monocytic leukemia cells. Toxicol. Appl. Pharmacol..

[41] Han W., Yang S., Xiao H., Wang M., Ye J., Cao L., Sun G. (2022). Role of Adiponectin in Cardiovascular Diseases Related to Glucose and Lipid Metabolism Disorders. Int. J. Mol. Sci..

[42] Li J., Yang Q., Liu H., Wang M., Pan C., Han L., Lan X. (2023). Phloretin alleviates palmitic acid-induced oxidative stress in HUVEC cells by suppressing the expression of LncBAG6-AS. Food Funct..

[43] Albers R.E., Kaufman M.R., Natale B.V., Keoni C., Kulkarni-Datar K., Min S., Williams C.R., Natale D.R.C., Brown T.L. (2019). Trophoblast-Specific Expression of Hif-1α Results in Preeclampsia-Like Symptoms and Fetal Growth Restriction. Sci. Rep..

[44] Matsuki M., Kabara M., Saito Y., Shimamura K., Minoshima A., Nishimura M., Aonuma T., Takehara N., Hasebe N., Kawabe J. (2015). Ninjurin1 is a novel factor to regulate angiogenesis through the function of pericytes. Circ. J. Off. J. Jpn. Circ. Soc..

[45] Madsen R.R., Semple R.K. (2022). PIK3CA-related overgrowth: Silver bullets from the cancer arsenal?. Trends Mol. Med..

[46] Arao Y., Korach K.S. (2021). The physiological role of estrogen receptor functional domains. Essays Biochem..

[47] Giguère V. (2018). Canonical signaling and nuclear activity of mTOR-a teamwork effort to regulate metabolism and cell growth. FEBS J..

[48] Strassheim D., Karoor V., Nijmeh H., Weston P., Lapel M., Schaack J., Sullivan T., Dempsey E.C., Stenmark K.R., Gerasimovskaya E. (2020). c-Jun, Foxo3a, and c-Myc Transcription Factors are Key Regulators of ATP-Mediated Angiogenic Responses in Pulmonary Artery Vasa Vasorum Endothelial Cells. Cells.

[49] Brouwer-Visser J., Huang G.S. (2015). IGF2 signaling and regulation in cancer. Cytokine Growth Factor Rev..

[50] Franklin S.L., Ferry R.J., Cohen P. (2003). Rapid insulin-like growth factor (IGF)-independent effects of IGF binding protein-3 on endothelial cell survival. J. Clin. Endocrinol. Metab..

[51] Ilari S., Nucera S., Passacatini L.C., Caminiti R., Mazza V., Macrì R., Serra M., Scarano F., Malafoglia V., Palma E. (2025). SIRT1: A likely key for future therapeutic strategies for pain management. Pharmacol. Res..

[52] Malkeyeva D., Киселева Е. (2016). The fuctional role of small heat shock protein Hsp67Bc in Drosophila melanogaster. Tsitologiia.

[53] Gao Q., Zhang L., Zhang B., Wang Q.-Y., Sun C.-F., Dong X.-T., Ying J. (2014). Phosphatase and tensin homolog overexpression decreases proliferation and invasion and increases apoptosis in oral squamous cell carcinoma cells. Oncol. Lett..

[54] Gaffen S.L. (2009). Structure and signalling in the IL-17 receptor family. Nat. Rev. Immunol..

[55] Karbach S., Croxford A.L., Oelze M., Schüler R., Minwegen D., Wegner J., Koukes L., Yogev N., Nikolaev A., Reißig S. (2014). Interleukin 17 drives vascular inflammation, endothelial dysfunction, and arterial hypertension in psoriasis-like skin disease. Arterioscler. Thromb. Vasc. Biol..

[56] Li Q., Ding S., Wang Y.M., Xu X., Shen Z., Fu R., Liu M., Hu C., Zhang C., Cao Q. (2017). Age-associated alteration in Th17 cell response is related to endothelial cell senescence and atherosclerotic cerebral infarction. Am. J. Transl. Res..

[57] van Dongen K.C.W., Linkens A.M.A., Wetzels S.M.W., Wouters K., Vanmierlo T., van de Waarenburg M.P.H., Scheijen J.L.J.M., de Vos W.M., Belzer C., Schalkwijk C.G. (2021). Dietary advanced glycation endproducts (AGEs) increase their concentration in plasma and tissues, result in inflammation and modulate gut microbial composition in mice; evidence for reversibility. Food Res. Int..

[58] Ott C., Jacobs K., Haucke E., Santos A.N., Grune T., Simm A. (2014). Role of advanced glycation end products in cellular signaling. Redox Biol..

[59] Forbes J.M., Cooper M.E. (2013). Mechanisms of diabetic complications. Physiol. Rev..

[60] Chen Z.-Q., Zhou Y., Chen F., Huang J.-W., Zheng J., Li H.-L., Li T., Li L. (2021). Breviscapine Pretreatment Inhibits Myocardial Inflammation and Apoptosis in Rats After Coronary Microembolization by Activating the PI3K/Akt/GSK-3β Signaling Pathway. Drug Des. Dev. Ther..

[61] Feng J., Xie L., Yu X., Liu C., Dong H., Lu W., Kong R. (2021). Perilipin 5 ameliorates high-glucose-induced podocyte injury via Akt/GSK-3β/Nrf2-mediated suppression of apoptosis, oxidative stress, and inflammation. Biochem. Biophys. Res. Commun..

[62] Li J., Wang T., Liu P., Yang F., Wang X., Zheng W., Sun W. (2021). Hesperetin ameliorates hepatic oxidative stress and inflammation via the PI3K/AKT-Nrf2-ARE pathway in oleic acid-induced HepG2 cells and a rat model of high-fat diet-induced NAFLD. Food Funct..

[63] Majumdar A.P.N., Du J. (2005). Phosphatidylinositol 3-kinase/Akt signaling stimulates colonic mucosal cell survival during aging. Am. J. Physiol. Gastrointest. Liver Physiol..

[64] Eo S.-H., Kim J., Kim S.J. (2015). Induction of G_2_/M Arrest by Berberine via Activation of PI3K/Akt and p38 in Human Chondrosarcoma Cell Line. Oncol. Res..

[65] Estrada-Gutiérrez G., Cappello R., Mishra N., Romero R., Strauss J.F., Walsh S.W. (2011). Increased expression of matrix metalloproteinase-1 in systemic vessels of preeclamptic women: A critical mediator of vascular dysfunction. Am. J. Pathol..

[66] Cambier S., Gouwy M., Proost P. (2023). The chemokines CXCL8 and CXCL12: Molecular and functional properties, role in disease and efforts towards pharmacological intervention. Cell. Mol. Immunol..

[67] Pardo A., Selman M. (2004). MMP-1: The elder of the family. Int. J. Biochem. Cell Biol..

[68] Bäck M., Ketelhuth D.F.J., Agewall S. (2010). Matrix metalloproteinases in atherothrombosis. Prog. Cardiovasc. Dis..

[69] Hanemaaijer R., Koolwijk P., Clercq L., de Vree W.J.A., van Hinsbergh V.W.M. (1993). Regulation of matrix metalloproteinase expression in human vein and microvascular endothelial cells. Effects of tumour necrosis factor alpha, interleukin 1 and phorbol ester. Biochem. J..

[70] Zhu Y., Hojo Y., Ikeda U., Takahashi M., Shimada K. (2000). Interaction between monocytes and vascular smooth muscle cells enhances matrix metalloproteinase-1 production. J. Cardiovasc. Pharmacol..

[71] Brinckerhoff C., Lynn M. (2002). Matrix metalloproteinases: A tail of a frog that became a prince. Mol. Cell Biol..

[72] Galis Z.S., Khatri J. (2002). Matrix metalloproteinases in vascular remodeling and atherogenesis: The good, the bad, and the ugly. Circ. Res..

[73] Newby A.C. (2004). Dual role of matrix metalloproteinases (matrixins) in intimal thickening and atherosclerotic plaque rupture. Physiol. Rev..

[74] Jacob M.P. (2003). Extracellular matrix remodeling and matrix metalloproteinases in the vascular wall during aging and in pathological conditions. Biomed. Pharmacother..

[75] Newby A.C., Johnson J.L. (2005). Genetic strategies to elucidate the roles of matrix metalloproteinases in atherosclerotic plaque growth and stability. Circ. Res..

[76] Higashikata T., Yamagishi M., Higashi T., Nagata I., Iihara K., Miyamoto S., Ishibashi-Ueda H., Nagaya N., Iwase T., Tomoike H. (2005). Altered expression balance of matrix metalloproteinases and their inhibitors in human carotid plaque disruption: Results of quantitative tissue analysis using real-time RT-PCR method. Atherosclerosis.

[77] Ye S., Galé C.R., Martyn C. (2003). Variation in the matrix metalloproteinase-1 gene and risk of coronary heart disease. Eur. Heart J..

[78] Struewing I.T., Durham S.N., Barnett C.D., Mao C.D. (2009). Enhanced endothelial cell senescence by lithium-induced matrix metalloproteinase-1 expression. J. Biol. Chem..

[79] Rawdanowicz T.J. (1994). Matrix metalloproteinase production by cultured human endometrial stromal cells: Identification of interstitial collagenase, gelatinase- A, gelatinase-B, and stromelysin-1 and their differential regulation by interleukin-1 alpha and tumor necrosis factor-alpha. J. Clin. Endocrinol. Metab..

[80] David-Dufilho M., Brussel E.M.-V., Topal G., Walch L., Brunet A., Rendu F. (2005). Endothelial thrombomodulin induces Ca^2+^ signals and nitric oxide synthesis through epidermal growth factor receptor kinase and calmodulin kinase II. J. Biol. Chem..

[81] Trivedi V., Boire A., Tchernychev B., Kaneider N.C., Leger A.J., O’Callaghan K., Covic L., Kuliopulos A. (2009). Platelet matrix metalloprotease-1 mediates thrombogenesis by activating PAR1 at a cryptic ligand site. Cell.

[82] Tressel S.L., Kaneider N.C., Kasuda S., Foley C., Koukos G., Austin K., Agarwal A., Covic L., Opal S.M., Kuliopulos A. (2011). A matrix metalloprotease-PAR1 system regulates vascular integrity, systemic inflammation and death in sepsis. EMBO Mol. Med..

[83] Nugent W.H., Mishra N., Strauss J.F., Walsh S.W. (2015). Matrix Metalloproteinase 1 Causes Vasoconstriction and Enhances Vessel Reactivity to Angiotensin II via Protease-Activated Receptor 1. Reprod. Sci..

[84] Wang M., Takagi G., Asai K., Resuello R.G., Natividad F.F., Vatner D.E., Vatner S.F., Lakatta E.G. (2003). Aging increases aortic MMP-2 activity and angiotensin II in nonhuman primates. Hypertension.

[85] Browatzki M., Larsen D., Pfeiffer C.A.H., Gehrke S.G., Schmidt J., Kranzhöfer A., Katus H.A., Kranzhöfer R. (2005). Angiotensin II stimulates matrix metalloproteinase secretion in human vascular smooth muscle cells via nuclear factor-kappaB and activator protein 1 in a redox-sensitive manner. J. Vasc. Res..

[86] Cui N., Hu M., Khalil R.A. (2017). Biochemical and biological attributes of matrix metalloproteinases. Prog. Mol. Biol. Transl. Sci..

[87] Wang C., Wen J., Zhou Y., Li L., Cui X., Wang J., Pan L., Ye Z., Liu P., Wu L. (2015). Apelin induces vascular smooth muscle cells migration via a PI3K/Akt/FoxO3a/MMP-2 pathway. Int. J. Biochem. Cell Biol..

[88] Xue J., Zhang Z., Sun Y., Jin D., Guo L., Li X., Zhao D., Feng X., Qi W., Zhu H. (2023). Research progress and molecular mechanisms of endothelial cells inflammation in vascular-related diseases. J. Inflamm. Res..

[89] Gomez D.E., Alonso D.F., Yoshiji H., Thorgeirsson U.P. (1997). Tissue inhibitors of metalloproteinases: Structure, regulation and biological functions. Eur. J. Cell Biol..

[90] Sun H.Y., Liu Y., Liu Q., Meng X.Q., Hao H. (2025). Study on the Mechanism of Shenfu Injection Regulating TIMP-3/MMP-1/syndecan-1Pathway to Improve Myocardial Injury in Rats with Sepsis. J. Li-Shizhen Tradit. Chin. Med..

[91] Baker A.H., Zaltsman A.B., George S.J., Newby A.C. (1998). Divergent effects of tissue inhibitor of metalloproteinase-1, -2, or -3 overexpression on rat vascular smooth muscle cell invasion, proliferation, and death in vitro. TIMP-3 promotes apoptosis. J. Clin. Invest..

[92] Shi L., Ji Q., Liu L., Shi Y., Lu Z., Ye J., Zeng T., Xue Y., Yang Z., Liu Y. (2020). IL-22 produced by Th22 cells aggravates atherosclerosis development in ApoE^−/−^ mice by enhancing DC-induced Th17 cell proliferation. J. Cell. Mol. Med..

[93] Del Pinto R., Ferri C. (2018). Inflammation-Accelerated Senescence and the Cardiovascular System: Mechanisms and Perspectives. Int. J. Mol. Sci..

[94] Harrington L.E., Hatton R.D., Mangan P.R., Turner H., Murphy T.L., Murphy K.M., Weaver C.T. (2005). Interleukin 17-producing CD4+ effector T cells develop via a lineage distinct from the T helper type 1 and 2 lineages. Nat. Immunol..

[95] Hernandez-Segura A., Brandenburg S., Demaria M. (2018). Induction and Validation of Cellular Senescence in Primary Human Cells. J. Vis. Exp..

[96] Feresin R.G., Huang J., Klarich D.S., Zhao Y., Pourafshar S., Arjmandi B.H., Salazar G. (2016). Blackberry, raspberry and black raspberry polyphenol extracts attenuate angiotensin II-induced senescence in vascular smooth muscle cells. Food Funct..

[97] Chien Y., Scuoppo C., Wang X., Fang X., Balgley B., Bolden J.E., Premsrirut P., Luo W., Chicas A., Lee C.S. (2011). Control of the senescence-associated secretory phenotype by NF-κB promotes senescence and enhances chemosensitivity. Genes Dev..

[98] Sun Y., Liu W.-Z., Liu T., Feng X., Yang N., Zhou H.-F. (2015). Signaling pathway of MAPK/ERK in cell proliferation, differentiation, migration, senescence and apoptosis. J. Recept. Signal Transduct. Res..

[99] Du S., Li Z., Xie X., Xu C., Shen X., Wang N., Shen Y. (2019). IL-17 stimulates the expression of CCL2 in cardiac myocytes via Act1/TRAF6/p38MAPK-dependent AP-1 activation. Scand. J. Immunol..

[100] Lei L., Zhao C., Qin F., He Z.-Y., Wang X., Zhong X.-N. (2016). Th17 cells and IL-17 promote the skin and lung inflammation and fibrosis process in a bleomycin-induced murine model of systemic sclerosis. Clin. Exp. Rheumatol..

[101] Xu X., Zhang W., Huang C., Li Y., Yu H., Wang Y., Duan J., Ling Y. (2012). A Novel Chemometric Method for the Prediction of Human Oral Bioavailability. Int. J. Mol. Sci..

[102] Chen L., Cao Y., Zhang H., Lv D., Zhao Y., Liu Y., Ye G., Chai Y. (2018). Network pharmacology-based strategy for predicting active ingredients and potential targets of Yangxinshi tablet for treating heart failure. J. Ethnopharmacol..

[103] Yang H., Zhang W., Huang C., Zhou W., Yao Y., Wang Z., Li Y., Xiao W., Wang Y. (2014). A novel Systems Pharmacology model for herbal medicine injection: A case using Reduning Injection. BMC Complement. Altern. Med..

[104] Liu Y., Yang X., Gan J., Chen S., Xiao Z.-X., Cao Y. (2022). CB-Dock2: Improved protein–ligand blind docking by integrating cavity detection, docking and homologous template fitting. Nucleic Acids Res..

[105] Jo S., Kim T., Iyer V.G., Im W. (2008). CHARMM-GUI: A web-based graphical user interface for CHARMM. J. Comput. Chem..

[106] Mark P., Nilsson L. (2001). Structure and Dynamics of the TIP3P, SPC, and SPC/E Water Models at 298 K. J. Phys. Chem. A.

